# microRNA 1307 Is a Potential Target for SARS-CoV-2 Infection: An *in Vitro* Model

**DOI:** 10.1021/acsomega.2c05245

**Published:** 2022-10-11

**Authors:** Elif Damla Arisan, D. Alwyn Dart, Guy H. Grant, Andrew Dalby, Derya Dilek Kancagi, Raife Dilek Turan, Bulut Yurtsever, Gozde Sir Karakus, Ercument Ovali, Sigrun Lange, Pinar Uysal-Onganer

**Affiliations:** † Gebze Technical University, Institute of Biotechnology, Gebze, Kocaeli 41400, Turkiye; ‡ Institute of Medical and Biomedical Education, St George’s University of London, Cranmer Terrace, Tooting, London SW17 0RE, United Kingdom; § School of Life Sciences, University of Bedfordshire, Park Square, Luton LU1 3JU, United Kingdom; ∥ School of Life Sciences, University of Westminster, London W1W 6UW, United Kingdom; ⊥ Acibadem Labcell Cellular Therapy Laboratory, İstanbul 34457, Turkiye; # Yeditepe University, Institute of Biotechnology, İstanbul 34755, Turkiye; ○ Tissue Architecture and Regeneration Research Group, School of Life Sciences, University of Westminster, London W1W 6UW, United Kingdom; ∇ Cancer Research Group, School of Life Sciences, University of Westminster, London W1W 6UW, United Kingdom

## Abstract

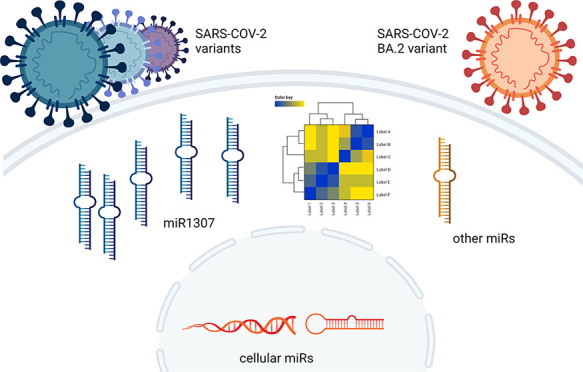

microRNAs (miRs) are proposed as critical molecular targets in SARS-CoV-2 infection. Our recent *in silico* studies identified seven SARS-CoV-2 specific miR-like sequences, which are highly conserved with humans, including miR-1307-3p, with critical roles in COVID-19. In this current study, Vero cells were infected with SARS-CoV-2, and miR expression profiles were thereafter confirmed by qRT-PCR. miR-1307-3p was the most highly expressed miR in the infected cells; we, therefore, transiently inhibited its expression in both infected and uninfected cells. The 3-(4,5-dimethylthiazol-2-yl)-2,5-diphenyl tetrazolium bromide (MTT) cell proliferation assay assessed cell viability following SARS-CoV-2 infection, identifying that miR-1307 expression is inversely correlated with cell viability. Lastly, changes in miR-1307-dependent pathways were analyzed through a detailed miRNOME and associated *in silico* analysis. In addition to our previously identified miRs, including miR-1307-3p, the upregulation of miR-193a-5p, miR-5100, and miR-23a-5p and downregulation of miR-130b-5p, miR34a-5p, miR-505-3p, miR181a-2-3p, miR-1271-5p, miR-598-3p, miR-34c-3p, and miR-129-5p were also established in Vero cells related to general lung disease-related genes following SARS-CoV-2 infection. Targeted anti-miR-1307-3p treatment rescued cell viability in infection when compared to SARS CoV-2 mediated cell cytotoxicity only. We furthermore identified by *in silico* analysis that miR-1307-3p is conserved in all SARS-CoV-2 sequences/strains, except in the BA.2 variant, possibly contributing to the lower disease severity of this variant, which warrants further investigation. Small RNA seq analysis was next used to evaluate alterations in the miRNOME, following miR-1307-3p manipulation, identifying critical pathobiological pathways linked to SARS-CoV-2 infection-mediated upregulation of this miR. On the basis of our findings, miRNAs like miR-1307-3p play a critical role in SARS-CoV-2 infection, including via effects on disease progression and severity.

## Introduction

The SARS-CoV-2 outbreak of respiratory illness, which causes coronavirus disease (COVID-19), also features as a multiorgan disease and was declared a pandemic by the World Health Organization (WHO) in March 2020.^1^ SARS-CoV-2 belongs to the family of coronaviruses, and among all the other seven coronaviruses, the b-subtype is the most fatal.^2,3^ SARS-CoV-2 enters the cell via binding of the spike (S) protein to the angiotensin-converting enzyme 2 (ACE2) receptor, activates innate and adaptive immune responses, and is accompanied by elevated inflammation markers (such as C-reactive protein (CRP), interleukin-2R (IL-2R), IL-6, IL-10, and TNFα).^4,5^ The variations between host responses to infection are unpredictable. For these reasons, there is a need to clarify the differences in virus and host-related molecular responses. The unresolved multifaceted molecular mechanisms underlying the plethora of clinical features caused by SARS-CoV-2 await further investigation.

microRNAs (miRs), which provide a fine-tuning mechanism for gene expression, could be essential biomarkers for predicting the severity of disease and mortality in SARS-CoV-2 infection.^6^ miRs are highly conserved noncoding RNAs of approximately 18–22 nucleotides in length and have gained increasing attention in SARS-CoV-2 and other viral infections. Viral miRNAs (v-miRs) can share common sequences with host miRs, which may provide interactions for immune evasion that have been reported between viruses and the miRs of host cells. There are three main proposed mechanisms: the first option is that RNA-based viral genomes may either avoid being targeted by the cellular miRs expressed against virus infection^7^ or block the cellular miRs to regulate essential proteins in the main signaling pathways.^8,9^ The second option is that viruses could synthesize their viral miRs to create a more favorable cellular environment to survive in the host cells.^10^ Third, viruses might manipulate cellular miRs to their advantage.^11,12^ According to recent reports, viral RNA may form complexes with host mRNAs and behave as miRs or rRNAs.^13,14^ Evidence also supports that the miR biogenesis by host cells may provide an efficient antiviral response.^15^ Several miRs have been identified as being differentially regulated in disease conditions and significantly correlate with elevated cytokine storms.^16^ For this reason, specific miRs, which are correlated to cell viability, could be used as disease biomarkers, to identify those at risk of cytokine storm, and possibly to act as targets for the treatment of COVID-19.^17^

Recent studies and our previous study have indicated that miR-1307-3p could be an essential regulator to trigger the production of various ILs and IL receptors in severe COVID-19 patients.^18−22^ We recently identified and reported several human miRs that show high sequence similarities to the SARS-CoV-2 genome.^18^ Importantly, the clinical samples assessed (BioProject data PRJNA615032) also confirmed the increased existence of miR-1307-3p in lung tissue samples following SARS-CoV-2 infection.^18^ We reported the significant sequence similarities between human miR-1307-3p and the SARS-CoV-2 genome, indicating the conservation of this sequence in most SARS-CoV-2 isolates obtained from different geographical regions based on *in silico* analysis.^18^ To further evaluate the role of miR-1307-3p in SARS-CoV-2 infection, we designed this current study in the presence of anti-miR-1307-3p in SARS-CoV-2 infected Vero cells and compared it to noninfected and infected only Vero cells. We confirmed several new miRNA targets in the experimentally infected Vero cells using RNaseq analysis. Additionally, our current study aimed to validate and further assess miR-1307 using *in vitro* cell systems and *in silico* analysis to identify the potential targets for miR-1307 that changed upon modulation and its possible roles in COVID-19-related disease pathology.

## Results and Discussion

This study first aimed to confirm the upregulation of the selected miRs (1307-3p, 8066 and 3611) following SARS-CoV-2 infection in Vero cells; next, we ran cell viability assays to screen for putative protective effects of anti-miR-1307 treatment against SARS-CoV-2 mediated cell viability loss. Lastly, we analyzed specific changes in miR-1307-dependent pathways through a detailed miRNOME and *in silico* analysis.

### miR-1307, miR-8066, and miR-3611 Expressions Are Significantly Elevated in SARS-CoV-2 Infected Cells

**Figure 1:**
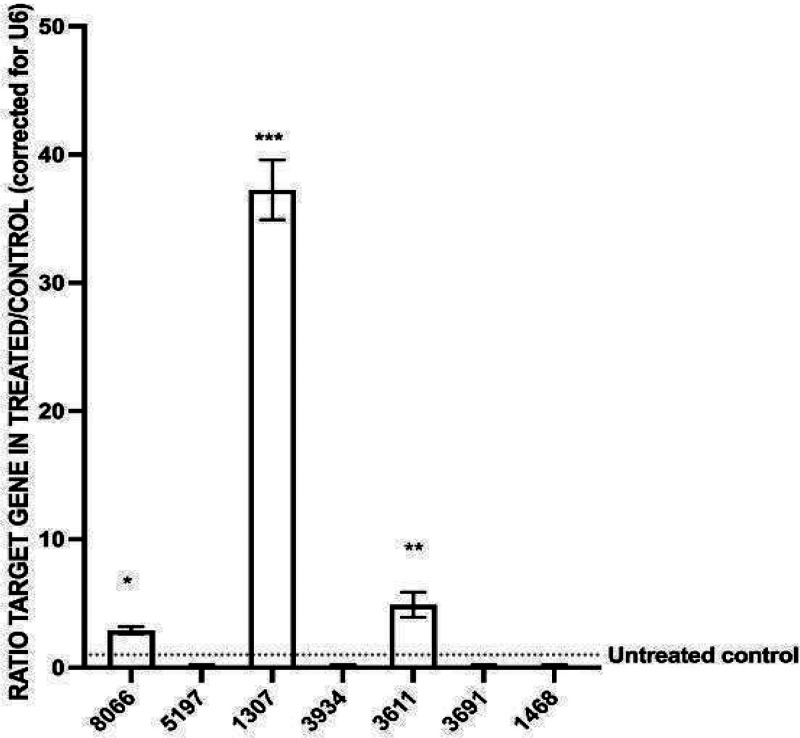
Expression levels of miR-8066, miR-5197, miR-1307, miR-3934, miR-3611, miR-3691, and miR-1498 in SARS-CoV-2 infected Vero cells compared to those in noninfected/control Vero cells as assessed by qRT-PCR. Significantly upregulated expression was observed for miR-1307 (38-fold), miR-3611 (4-fold), and miR-8066 (2-fold). The column graphs represent the average of three technical replicates of RNA isolated from three experimental replicates. Data normalized according to RNU6 expression by fold analysis (*n* = 3, *p* < 0.05 for all). *p*-values are indicated as *p ≤ 0.05 (for miR-8066); **p ≤ 0.01 (for miR-1307); ***p ≤ 0.001 (for miR-3611); ****p ≤ 0.0001); error bars indicate standard deviation (SD).

Following isolation of the SARS-CoV-2 Wuhan strain variant from a COVID-19 patient, as previously described,^23^ we determined the CPE and TCID50 dose of the propagated virus *in vitro* in preparation for the inoculation assay with miR inhibitors. Briefly, Vero cells were inoculated with the SARS-CoV-2 in a dose-dependent manner; when 100% CPE of the virus was reached, the TCID50 dose was achieved in 10^–3^ dilution of the SARS-CoV-2 virus. We then determined the concentration of the virus as 1 × 10^6^ TCID50/mL (Figure S1A). Next, the SARS-CoV-2 RNA copy number was calculated as 4.5 × 10^17^/mL in the sample with a serial dilution using qRT-PCR with E, N, and Orf1ab gene-specific primers (Figure S1B,C). These results enabled the preparation of the dose-determined SARS-CoV-2 virus in further experiments. The expression levels of our seven previously reported SARS-CoV-2 related miRs (8066, 5197, 1307, 3934, 3611, 3691, 1468) [18] were quantified in the SARS-CoV-2 infected Vero cells. The expression of miR-1307 was found to be the most significantly increased miR out of these targets in the infected cells (38-fold increase; *n* = 3; *p* < 0.0001; Figure 1), followed by miR-3611 (4-fold increase; *n* = 3; *p* < 0.0001; Figure 1) and miR-8066 (2-fold increase; *n* = 3; *p* < 0.0001; Figure 1), compared with that in the control/uninfected Vero cells.

We have previously reported that the following SARS-CoV-2 miR expression levels changed in infected Calu-3, A549, and NHEJ cell models and clinical samples.^18^ We found sequence similarities with miRs in the SARS-CoV-2 genome, which are conserved in different variants except BA.2. These findings proposed that a functional role of the virus genome might lead to the generation of crucial miRs linked to the severity of diseases. In a similar vein, it is known that miRs are widely recognized as a novel and stable biomarker for diagnosis in several pathologies, including in infectious diseases, where recent reports emphasize that miR expression patterns can be utilized to predict the severity of viral infections,^24,25^ including in COVID-19.^6^ miR profiling and prediction associated with SARS-CoV-2 infection were reported in previous studies by our group and others.^6,18,26−30^ miR-1307 has been identified as one of the crucial targets in SARS-CoV-2 mediated cellular response pathways. miR-1307-3p involves TGF-β signaling as the signature of inflammatory response oxygen dependency, persistent wheezing, and chronic lung diseases.^18^

### miR-1307 Mimic Sequences Show Variations between SARS-CoV-2 Isolates

To identify whether miR-1307-3p is present within different SARS-CoV-2 variants, we used multiple sequence alignments of this miR motif against the complete genome sequences of most known human coronaviruses (Table 2). This included the WH-1 SARS-CoV-2 reference sequence, the current variants of concern (Alpha, Beta, Gamma, Delta, and Omicron BA.1 and BA.2), SARS, MERS, four “cold-like” viruses (NL63, 229E, OC43, and HKU1), and the closely related bat coronavirus RatG13. The region assessed was aligned with miR-1307-3p, which seems to be fully conserved in WH-1, RatG13, and four of the concern variants (VoC’s), Alpha, Beta, Gamma, and Omicron BA.1, while there is a single, different mutation in each Delta and SARS. Notably, a 26-nucleotide gap in this region in the BA.2 variant closely corresponds to gaps in NL63 and 229E, cold viruses. An identical gap is also present in the BA.2 lineage variants, BA.2.75, BA.4, and BA.5 (data not shown). It is also part of a long gap in MERS and HKU1, while only the first seven nucleotides are aligned in OC43. Moreover, we found that the region where the miR-1307-3p motif is based in human coronaviruses is fully conserved in four VOCs, Alpha, Beta, Gamma, and Omicron BA.1, while there is a single, different mutation in each Delta and SARS (Table 2). However, a 26-nucleotide gap corresponding to a putative 3′UTR stem-loop is found in this region in the BA.2 variant, closely corresponding to gaps in NL63 and 229E, cold viruses. It is also part of a long gap in MERS and HKU1, while only the first seven nucleotides are aligned in OC43. This suggests that the proposed participation of this region in the replication mechanism of beta coronaviruses cannot be universal. In this sequence region, it appears to be more similar to the cold viruses than to the other SARS-CoV-2 sequences. Similarly, our previous comparative *in silico* analysis showed that miR-1307 was highly conserved in pangolin, pig, cow, bat, and humans, indicating that this miR may play a role in a putative initial zoonotic transmission.^31^ Importantly, also, the alignment of miR-1307-3p against both the RatG13 bat virus and the Wuhan strain demonstrates complete conservation of this sequence during a possible zoonotic transfer and suggests that the loss of miR-1307-3p in the SARS-CoV-2 BA.2 variant in humans could represent a change in the SARS-CoV-2 pathogenicity (Table 1). The recent findings suggest that genomic SARS-CoV-2 mutations influence miR expression levels, leading to clinical response variations. The recent mutant type of SARS-CoV-2 might induce a lower immune response and a weaker cytokine storm.^18,32−34^

**Table 1:**
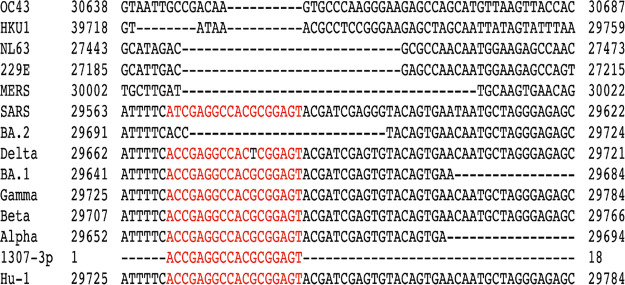
A Segment of the Complete Genomic Alignment of SARS-CoV-2 Variants of Concern (Alpha, Beta, Gamma, Delta, and Omicron BA.1 and BA.2), the Original Wuhan Reference Sequence (WH-1), and Other Human Coronaviruses with miR-1307-3p^a^

a Findings highlight the conservation of miR-1307 in most SARS-CoV-2 sequences, except the BA.2 variant, similar to that seen for the cold viruses. Accession codes are given in Table S1.

### miR-1307 Expression Modulates SARS-CoV-2-Dependent Cell Survival

**Figure 2:**
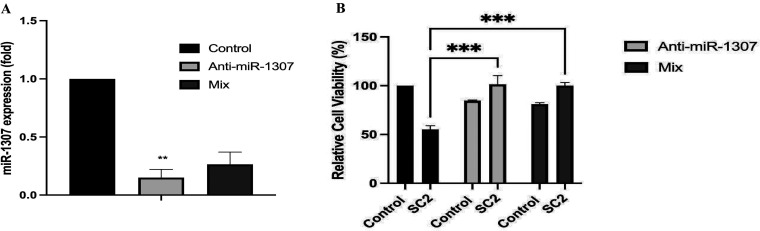
Downregulation of miR-1307 alone (anti-miR-1307) or combined with anti-miRs 8066 and 3611 (mix) with reversed cell proliferation in SARS-CoV-2 infected Vero cells. (A) Anti-miR-1307 transfection reduced the miR-1307 expression level in SARS-CoV-2 infected cells. (B) Effects of miR inhibition on cell survival were investigated following SARS-CoV-2 infection. SARS-CoV-2 infection alone decreases cell proliferation, whereas inhibition of miR-1307 alone (Anti-miR-1307) or in combination with anti-miRs 8066 and 3611 (mix; each anti-miR at 100 nM concentration) rescues cell proliferation (*n* = 3). *p*-values are indicated on the graph; the Bonferroni two-way ANOVA test was used to determine the significance level.

Our *in vitro* experimental model in this study confirmed the upregulation of several miRs previously identified as miR-1307, miR-8066, and miR-3611 following SARS-CoV-2 infection (Figure 1). Therefore, we transiently transfected cells with anti-miR-1307-3p or mixed anti-miRs against miR-1307, miR-8066, and miR-3611 to evaluate the virus-mediated cytotoxicity and changes in noncoding RNAs to clarify altered pathways due to the diminished miR-1307-3p presence in SARS-CoV-2 infected Vero cells. To optimize anti-miR-1307 treatment, we first confirmed the expression levels of miR-1307 in Vero cells. To increase the efficiency of anti-miR mediated responses, we also assessed the treatment of anti-miRs against the upregulated miR-1307, miR-8066, and miR-3611 following SARS-CoV-2 infection. According to our findings in Figure 2A, the miR-1307 expression profile significantly downregulated following anti-miR-1307 alone or the combined anti-miR treatment in Vero cells. Following confirmation of the selected 100 nM anti-miR treatment being efficient, we ran the experiments with SARS-CoV-2 infection, which led to a 40% decrease in Vero cell proliferation (Figure 2B), whereas targeted inhibition of miR-1307 (and the anti-miR combined treatment against three upregulated miRs) resulted in a significant 24% and 16% increase in cell survival/proliferation in SARS-CoV-2 infected Vero cells, respectively (*n* = 3; *p* < 0.001, Figure 2B). Following targeted inhibition of miR-1307, increased cell viability was observed in response to SARS-CoV-2 infection, indicating an essential role in SARS-CoV-2-related pathology. Thus, we concluded that suppression of miR-1307 prevented the cytotoxic effects of viral infection. Recently, it has been noted that hsa-miR-1307-3p can inhibit clathrin-dependent endocytosis by inhibiting AP-2 and PIP5K and might suppress exocytosis by inhibiting actin.^35^ Therefore, controlling these pathways by hsa-miR-1307-3p could be an effective strategy for SARS-CoV-2 infection. SARS-CoV-2 mediated miR-1307-3p expression was reduced after using its specific inhibitor, which leads to rescue of cell survival after infection (Figure 2). Notably, the single anti-miR-1307-3p treatment was more effective than a mixed treatment.

### SARS-CoV-2 Infection Globally Altered miRs Related to Infection in Vero Cells

We next analyzed the effects of SARS-CoV-2 infection on the global expression of miRs and assessed the specific downstream effects of miR-1307 inhibition. We used RNA-seq analysis to evaluate the small RNA species, including mature miRs. We compared the expression of the miRs in SARS-CoV-2 infected vs uninfected Vero cells. We also inhibited miR-1307-3p by using anti-miR-1307 and then examined the detailed miRNOME analysis to evaluate possible changes in cell signaling pathways.

**Figure 3:**
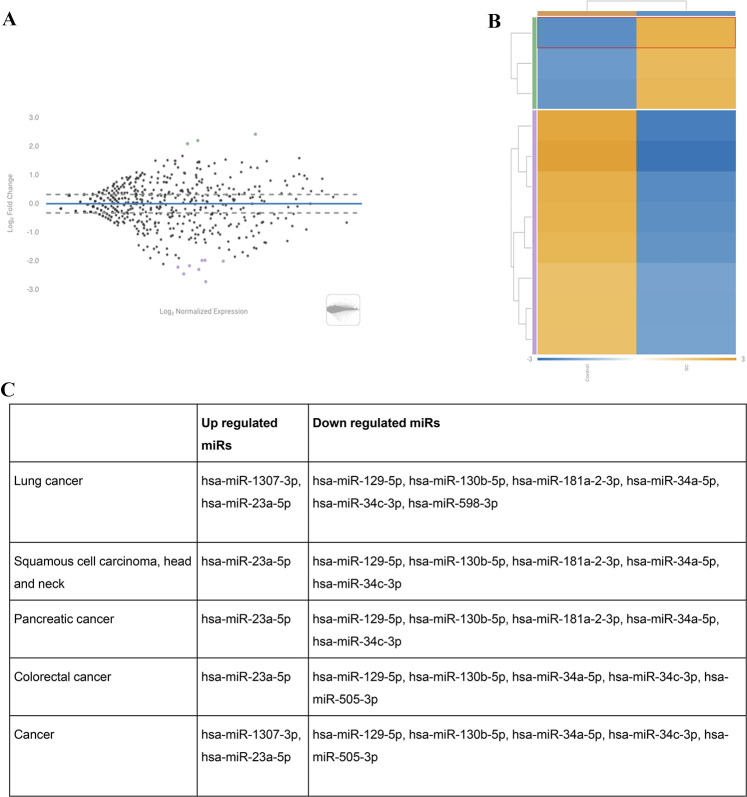
(A) Volcano plot showing differentially expressed miRs from SARS-CoV-2 infected Vero cells compared to uninfected control cells. (B) Heat map comparison for the most altered top miRs in uninfected control and SARS-CoV-2 (SC) cells. (C) Pathologies related to the main upregulated and downregulated miRNA targets.

Aligning the small RNA libraries using Refseq gene annotation showed that 2652 miRNA targets were altered in SARS-CoV-2 infected cells compared to uninfected Vero cells and control groups of the Vero samples (Figure 3A). The most significantly upregulated miRNA targets included hsa-miR-5100, hsa-miR-1307-3p, and hsa-miR-23a-5p, while the most significantly downregulated targets included hsa-miR-129-5p, hsa-miR-1271-3p, hsa-miR-130b-5p, hsa-miR-181a-2-3p, hsa-miR-34a-5p, hsa-miR-34c-3p, hsa-miR-598-3p, and hsa-miR-505-3p. All these miRNA targets were furthermore shown to be associated with different pathological pathways, including lung cancer (Figure 3C). It was shown that malignancies such as squamous cell carcinoma, head and neck cancer, pancreatic cancer, and colorectal cancer were also linked to altered miRNOME due to SARS-CoV-2 infection. Previous studies highlighted viral infection-related roles of miR-1307, for instance, a novel human miR upregulated in Epstein–Barr virus (EBV)-positive nasopharyngeal carcinomas.^36^ Bavagnoli et al. reported that miR-1307 is vital in regulating viral replication in the influenza A virus H1N1.^37^ In our study, we found that miR-1307-3p is one of the top leading targets within several 1872-pooled miRs. It has the highest affinity to the SARS-CoV-2 genome and may prevent GRP78 production and modulate Bcl-2 expression.^15^ The upregulation of miR-1307-3p upon SARS-CoV-2 infection was shown in another study using the human Calu-3 cell line (GSE148729).^28^ Another *in silico* study showed that the SARS-CoV-2 genome has various potential miR-binding sites within the 5′ and 3′ UTRs, which can interact with host cellular noncoding RNAs, including miR-1307-3p.^30^ miR-1307-3p may bind on two potential sites within the SARS-CoV-2 s2m element, a highly conserved 41-nucleotide element among coronaviruses and other viral families.^38^ Experimental models confirmed that miR-1307-3p could bind to SARS-CoV-2 to provide a beneficial output on the viral life cycle.^38^ In the current study, we propose other experimental evidence to clarify the role of miR-1307-3p on SARS-CoV-2 infection. Therefore, as stated in previous findings, we proposed that hsa-miR-1307-3p could be a critical target for preventing or controlling SARS-CoV-2 infection.^18,39^ However, this preventive role in viral replication has also been linked with a mutation found in the 3′ UTR region of the target site.^21^

**Figure 4:**
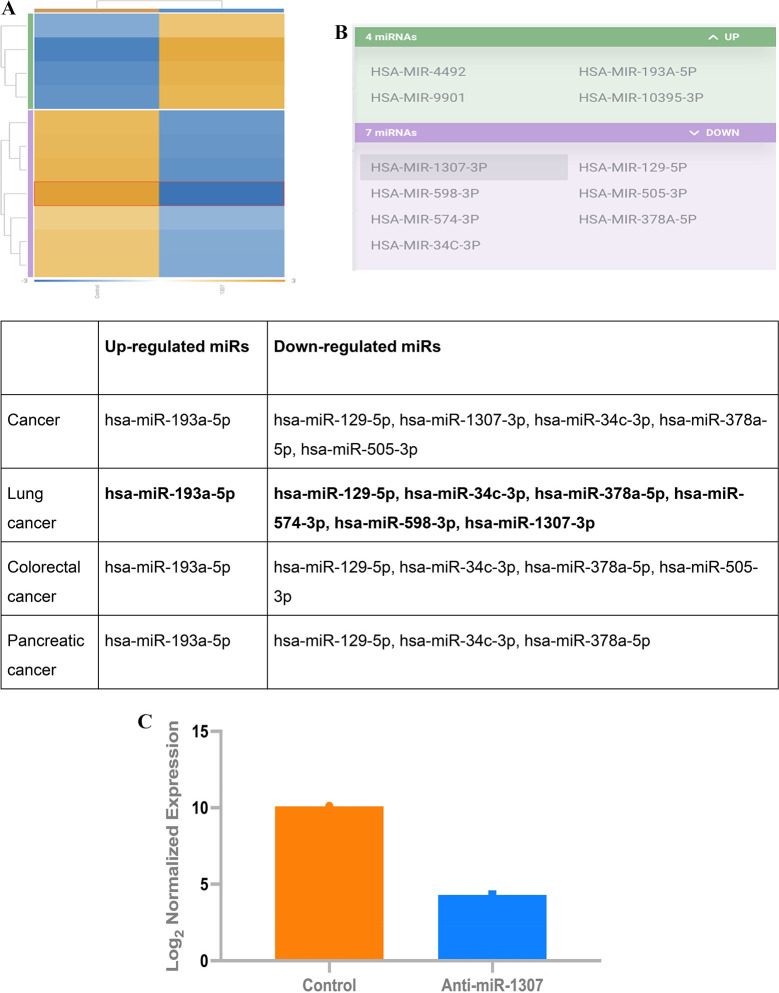
(A) Volcano plot displaying 485 differentially expressed miRs from control vs anti-miR-1307 transfected Vero cells. According to significantly altered miRNA targets, 4 miRNAs are upregulated and 7 miRNAs are downregulated. (B) Upregulated and downregulated miRNA targets affected different diseases. (C) The expression level of miR-1307-3p following anti-miR-1307 treatment in Vero cells according to the analysis software of ROSALIND.

We also assessed the profile of altered miRs following the anti-miR-1307 treatment in Vero uninfected cells (Figure 4). According to the volcano plot, 485 miRs were altered following miR-1307-3p inhibition. As shown in Figure 4A, miR-4492, miR-9901, miR-193a-5p, and miR-10395-3p were upregulated and miR-13073p, miR-598-3p, miR-574-3p, miR-34c-3p, miR-129-5p, miR-505-3p, and miR-378a-5p were downregulated following anti-miR-1307 treatment. These altered miRs were related to a number of cancers, including lung cancer (Figure 4B). Anti-miR-1307 treatment in Vero cells led to significant downregulation of this miR by 5.8-fold compared to uninfected Vero cells (Figure 4C). miR-1307 inhibition of the uninfected cells in the current study showed deregulations of several miRs’ expressions. While miR-4492, miR-9901, miR-193a-5p, and miR-10395-3p were upregulated, miR-1307-3p, miR-598-3p, miR-574-3p, miR-34c-3p, miR-129-5p, miR-505-3p, and miR-378a-5p were significantly downregulated following anti-miR-1307 treatment (Figure 4). All these miR targets were linked to lung, colorectal, and pancreatic carcinomas, while miR-9901 and miR-10395-3p were not linked to any known target within microT-CDS, Targetscan, and TarBase. However, it must also be noted that such *in silico* analysis can only include reported data, and hence, any hitherto unknown disease relations remain to be understood. In a recent study, miR-10395-3p was shown to be a significant liquid biopsy target in HIV/HCV positive patients.^40^ Additionally, KEGG and GO pathway analysis of all altered miRs following anti-miR-1307 treatment, except miR-9901 and miR-10395-3p, showed the potential relationship with axon guidance (hsa04360), the ErbB signaling pathway (hsa04012), metabolism of xenobiotics by cytochrome P450 (hsa00980), nicotine addiction (hsa05033), morphine addiction (hsa05032), cell adhesion molecules (CAMs) (hsa04514), GABAergic synapse (hsa04727), adrenergic signaling in cardiomyocytes (hsa04261), and mucin type O-glycan biosynthesis (hsa00512). GO pathway analysis showed nucleic acid binding transcription factor activity (GO:0001071), the cellular protein modification process (GO:0006464), the cellular nitrogen compound metabolic process (GO:0034641), ion binding (GO:0043167), and the biosynthetic process (GO:0009058) (Figure S2). The upregulation of miR-23a-3p was significant in our study following SARS-CoV-2 infection by previous reports, which indicates that early cases can be diagnosed with miR-23a-3p.^41^ The most significant target following anti-miR-1307 treatment was hsa-miR-34c-3p, which has been reported to be highly elevated in serum samples using next-generation sequencing (GSE182183) following SARS-CoV-2 infection.^42^ In the same study, hsa-miR-193a-5p was linked to ventilation requirements in the severe stages of SARS-CoV-2 infection. Previous *in silico* analysis showed that hsa-miR-193a-3p is the predicted and validated target of miR-1307.^18^ Additionally, the predicted targets of hsa-miR-1307-3p include miR-193b-3p, hsa-miR-222-3p, hsa-miR-615-3p, hsa-miR-221-3p, hsa-miR-744-5p, hsa-miR-3190-3p, hsa-miR-423-3p, hsa-miR-326, hsa-miR-484, hsa-miR-325, hsa-miR-326, hsa-miR-605-5p, hsa-miR-92a-3p, hsa-miR-193b-3p, and hsa-miR-4743-5p, precatalytic spliceosome, U12-type spliceosomal complex, and the structural constituent of the postsynaptic actin cytoskeleton. These pathways were similar to previous KEGG and GO predictions, which may be critical to the clarification of viral infection-related miR-1307-3p upregulation, which was related to different miRs (Figure S3). For this purpose, further experiments will be critical to evaluate the relationship between miR-1307-3p and its predicted targets.

**Figure 5:**
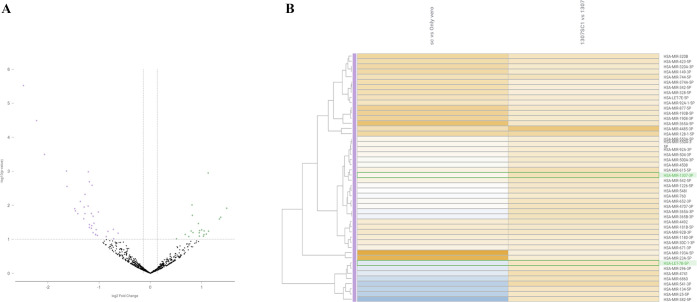
(A) miR-1307 was highly upregulated following SARS-CoV-2 infection. According to ROSALIND *in silico* RNaseq analysis, differently upregulated and downregulated miRNA targets following treatment were shown in Group 1 (SARS-CoV-2 infected vs Vero cells) and Group 2 (anti-miR-1307 treated SARS-CoV-2 infected cells vs anti-miR-1307 treated Vero cells). The color scheme indicates that blue is downregulated and orange is upregulated. (B) Venn scheme is drawn to compare miRs obtained from the top altered miRNA expression profile set. Control Vero cells; SC+: SARS-CoV-2 infected Vero cells; anti-miR-1307: anti-miR-1307 treated control cells.

**Figure 6:**
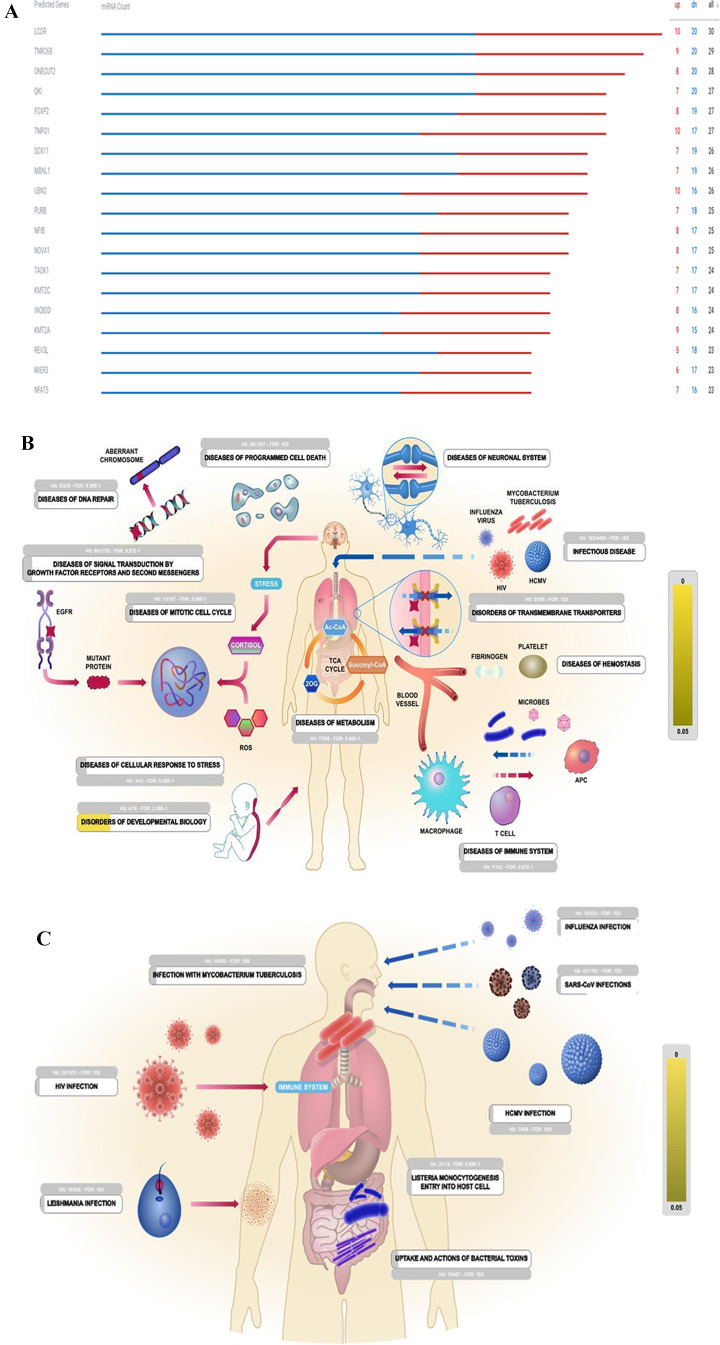

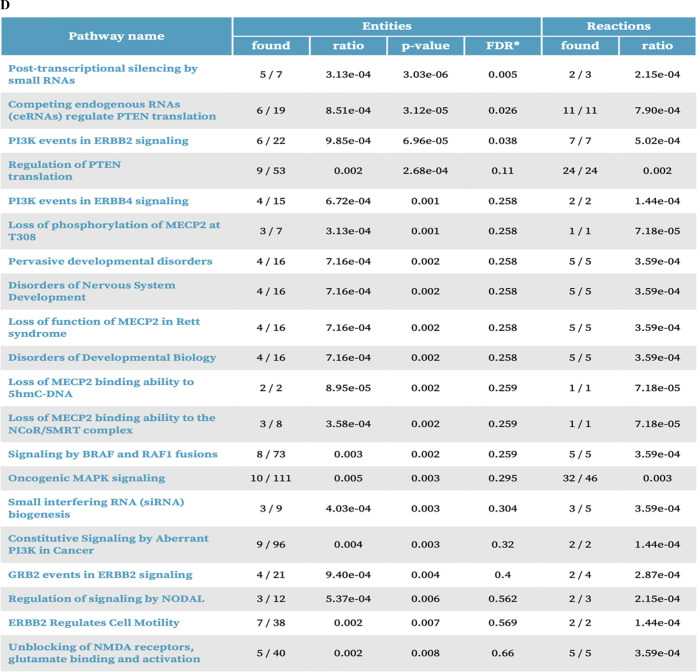
(A) The top altered validated miRNA targets are shown according to ROSALIND analysis for the comparison of anti-miR-1307 treated SARS-CoV-2 infected cells compared to untreated SARS-CoV-2 infected cells. (B) The pathway analysis is shown according to Reactome for the validated miRNA targets. The illustration is taken from Reactome data analysis report. (C) The top 25 altered pathways for the validated miRNA targets in Reactome analysis. The illustration is taken from Reactome data analysis report. (D) The leading pathways according to the ROSALIND pathway analysis tool is shown for the most altered miRs between Group 1 and Group 2. The illustration is taken from Reactome data analysis report.

According to the volcano plot, there were 56 significantly differently regulated miRs between Group 1 (SARS-CoV-2 infected vs Vero cells) and Group 2 (anti-miR-1307 treated SARS-CoV-2 infected cells vs anti-miR-1307 treated Vero cells) (Table 2 and Figure 5). SARS-CoV-2 mediated miR-1307-3p upregulation was suppressed with anti-miR-1307 treatment. This alteration led to changes in the different miRs, such as Let7b (Figure 5A,B). Briefly, the most remarkable change in miR targets was identified in comparison between Group 1 (SARS-CoV-2 infected vs Vero cells) and Group 2 (anti-miR-1307 treated SARS-CoV-2 infected cells vs anti-miR-1307 treated Vero cells) (Figures 5, 6, S4, and S5). Verified pathway analysis highlighted infectious diseases such as influenza, tuberculosis, and HIV. Infectious diseases are overrepresented as the third group in the Reactome analysis for all upregulated and downregulated miRs between Group 1 and Group 2.

**Table 2:** 56 Significantly Differently Regulated miRs Were Detected between Group 1 (SARS-CoV-2 Infected vs Vero Cells) and Group 2 (Anti-miR-1307 Treated SARS-CoV-2 Infected Cells vs Anti-miR-1307 Treated Vero Cells)

comparison groups	upregulated miRs	downregulated miRs
**Group 1** (SARS-CoV-2 infected vs Vero cells) vs **Group 2** (anti-miR-1307 treated SARS-CoV-2 infected cells vs anti-miR-1307 treated Vero cells)	hsa-miR-5698, hsa-miR-550a-3-5p, hsa-miR-550a-5p, hsa-miR-1307-3p, hsa-miR-6724-5p, hsa-miR-342-5p, hsa-miR-1234-3p, hsa-miR-222-5p, hsa-miR-92b-5p, hsa-miR-500b-5p, hsa-miR-27b-5p, hsa-miR-365a-5p, hsa-miR-219a-1-3p, hsa-miR-23a-5p, hsa-miR-1908-5p, hsa-miR-128-1-5p, hsa-Let-7e-5p, hsa-miR-744-5p, hsa-miR-320a-3p, hsa-miR-193b-5p, hsa-miR-423-5p, hsa-miR-103a-3p	hsa-miR-1271-5p, hsa-miR-130B-5p, hsa-miR-598-3p, hsa-miR-485-3p, hsa-miR-455-5p, hsa-miR-505-3p, hsa-miR-4768-5p, hsa-miR-6529-3p, hsa-miR-425-3p,hsa-miR-543, hsa-miR-181A-2-3p, hsa-miR-34A-5p, hsa-miR-654-3p, hsa-miR-487B-3p, hsa-miR-99B-5p, hsa-miR-148B-3p, hsa-miR-539-5p, hsa-miR-29A-5p, hsa-miR-34C-3p, hsa-miR-205-5p, hsa-miR-323B-3p, hsa-miR-363-3p, hsa-miR-550A-3p, hsa-miR-409-3p, hsa-miR-139-5p, hsa-miR-10399-3p, hsa-miR-2355-3p, hsa-miR-129-5p, hsa-miR-191-3p, hsa-miR-34B-3p, hsa-miR-574-3p, hsa-miR-221-3p,hsa-miR-25-3p, hsa-miR-18A-3p, hsa-miR-27A-3p

Biological processes were captured in Reactome analysis by identifying the molecules (DNA, RNA, protein, small molecules) and describing the details of their interactions. For this purpose, we first identified the most altered representative target genes according to downregulated and upregulated miRs (Figure 6A). From this molecular viewpoint, human disease pathways were identified with three mechanistic causes: the inclusion of microbially expressed proteins, altered functions of human proteins, or changed expression levels of otherwise functionally normal human proteins (Figure 6B). The first group encompasses infectious diseases such as influenza, tuberculosis, and HIV. The second group involves human proteins modified by a mutation or an abnormal post-translational event that produces an aberrant protein by a novel function. Infectious diseases are represented as the third group in Reactome analysis as microbial–human protein interactions and the consequent events. Depending on the biological pathway/process immediately affected by altered gene expression profiles due to changes in miR expression levels and variants, Reactome analysis highlighted the different pathways, including the infectious disease category. Processes annotated in this category (R-HSA-5663205) included the life cycles of SARS-CoV viruses, influenza virus, and HIV (human immunodeficiency virus), some metabolic processes mediated by intracellular *Mycobacterium tuberculosis*, the actions of clostridial, anthrax, and diphtheria toxins, and the entry of *Listeria monocytogenes* into human cells. The other signal transduction-related categories were growth factor receptors and second messengers, diseases of the mitotic cell cycle, diseases of the cellular response to stress, diseases of programmed cell death, diseases of DNA repair, disorders of transmembrane transporters, diseases of metabolism, diseases of the immune system, diseases of the neuronal system, disorders of developmental biology, disorders of extracellular matrix organization, and diseases of hemostasis (Figure 6C). The most altered three leading pathways were post-transcriptional silencing by small RNAs, competing for endogenous RNAs, and PI3K events in ERBB2 signaling according to the ROSALIND pathway analysis tool between Group 1 and Group 2 (Figures 6D, S4, and S5).

## Conclusions

In summary, we report that miRs with acute effects on physiological and pathobiological pathways were modulated in SARS-CoV-2 infected Vero cells, specifically, in response to miR-1307 inhibition, which significantly affected viral infection ability *in vitro*. These current findings add to the increasing evidence for critical roles of miRs in SARS-CoV-2 infection, zoonotic transmission, and viral–host coevolution, including modulatory responses on host immune responses and effects on patient outcomes and comorbidities. The current study highlights the potential roles of selected miRs as targets in SARS-CoV-2 treatment strategies.

On the basis of the findings of this current study, we suggest that miR-1307-3p may be an important biomarker for SARS-CoV-2 infection and disease severity. It has been suggested that miR-1307-3p could be involved in the viral entry of SARS-CoV-2 and propagation. Therefore, its expression levels may predict the outcome of the host immune response. Overall, the SARS-CoV-2 mediated modulation of miR-1307 could lead to a range of altered cell responses involved in pathobiological processes. For this reason, the observed loss of miR-1307-3p in the SARS-CoV-2 BA.2 strain in humans may be worthy of note. Our data presented here suggest that the elevation of miR-1307-3p following SARS-CoV-2 infection plays a crucial role in cell survival and that inhibiting miR-1307 reduces viral infection-induced cell death and may affect downstream pathological responses.

## Methods

### Cell Culture and Virus Propagation

The current study used the SARS-CoV-2 Wuhan strain. The virus propagation process was performed using the Vero cell line (CCl-81, ATCC, Manassas, Virginia USA) as described in ref (23). The cell coculture was suspended in media composed of DMEM with high glucose (Thermo Fisher Scientific, USA), 2% fetal bovine serum (Thermo Fisher Scientific, USA), and 1% penicillin–streptomycin–amphotericin (PSA) solution (Pan Biotech, Germany). For transduction, 100 000 cells were seeded into each well of 96 well plates. The cytopathic effect resulting from SARS-CoV-2 host cell lysis was recorded under a real-time cell analysis device (xCELLigence, Roche, USA). Vero cells (2.5 × 10^4^ cells/well) were incubated in gold microelectrode embedded microtiter wells in the Real-Time Cell Analysis instrument (xCELLigence, Roche, USA) at 37 °C for 24 h to normalize the cell index with coated cells. Then, SARS-CoV-2 virus, in a serial dilution (10^–1^ to 10^–6^), was inoculated into the Vero cells, which were analyzed in real-time for 96 h. Cell analysis was normalized to the 24 h culture value before incubation. A normalized cell index was used to show the proliferation and viability of the adherent cells (the higher cell index means the higher viability and proliferation). As the cell index value decreases, an increase in the cytopathic effect (CPE) of the virus was observed compared with that of the untreated control Vero cells.

### Genomic Characterization and Quantitative Titration of SARS-CoV-2

SARS-CoV-2 specific qRT-PCR was performed using the BOSPHORE Novel Coronavirus (2019-nCoV) Detection Kit (Anatolia Gene works, Istanbul, Turkey) along with Orf1ab and E gene primers (Sentromer DNA Technologies, Istanbul, Turkey). Total RNA isolations were performed from the SARS-CoV-2 specimens using Direct-Zol RNA Miniprep Kits (Zymo Research, Irvine, USA). qRT-PCR was performed with the Quantivirus SARS-CoV-2 Test Kit (Diacarta, California, USA) according to the manufacturer’s protocol.

### RNA Isolation and qRT-PCR for Assessing Selected miRs

Total RNA isolation from SARS-CoV-2 infected and control cells was performed as described above. According to the manufacturer’s instructions, RNA was reverse transcribed to cDNA using the qScript microRNA cDNA synthesis kit (Quantabio, Lutterworth, UK), and selected miRs were assessed by qPCR using the PerfeCTa SYBR Green SuperMix (Quantabio, Anatolia GeneWorks Istanbul, Turkey). RNU6 was used as a normalization reference RNA using the comparative cycle threshold method.^43^ Primers for each miR assessed included hsa-miR-8066, -5197, -3611, -3934-3p, -1307-3p, -3691-3p, and -1468-5p (Table 3; Sentromer DNA Technologies, Istanbul, Turkey). The following thermocycling conditions were used: denaturation at 95 °C for 2 min, then 40 cycles of 95 °C for 2 s, 60 °C for 15 s, and extension at 72 °C for 15 s.

**Table 3:** Sequences of the Primers Were Used to Detect the Seven Individual SARS-CoV-2 Related miRs (hsa-miR-8066, -5197, -3611, -3934-3p, -1307-3p, -3691-3p, and -1468-5p) by qRT-PCR

miRs	primer sequences
hsa-miR-8066	CAA TGT GAT CTT TTG GAT GTA
hsa-miR-5197	AAG AAG AGA CTG AGT CAT CGA AT
hsa-miR-3611	TTG TGA AGA AAG AAA TTC TTA
hsa-miR-3934	TGC TCA GGT TGC ACA GCT GGG A
hsa-miR-1307	CGG CGT GGC GTC GGT CGT G
hsa-miR-3691	AGT GGA TGA TGG AGA CTC GGT AC
hsa-miR-1468	AGT GGA TGA TGG AGA CTC GGT AC
RNU6	forward 5′-GCTTCGGCAGCACATATACTAAAAT-3′, reverse 5′-CGCTTCACGAATTTGCGTGTCAT-3′

### Transfection of Vero Cells Using Anti-miRs

On the basis of the experimental outcomes of the miR expression profile analysis in Vero cells in the current study, the notably highly expressed miR-1307 was selected for further targeted inhibition experiments. Anti-miR-1307 treatment alone or in combination with the anti-miR-3611 and anti-miR-8066 (mix-100 nM concentrations) treatment from IDT (Integrated DNA Technologies, Leuven, Belgium) was performed on the Vero cells, which were infected with SARS-CoV-2 (amount of virus used: 100 TCID50 SARS-CoV-2). Briefly, anti-miR transiently transfected cells were seeded at 1 × 10^4^ density for 72 h and then used for further experiments detailed below. Vero cells were seeded in a 96-well plate at a density of 1 × 10^4^ cells per well. Upon cells reaching 70–80% confluency, the miR-1307 inhibitor transfection was performed 24 h before virus infection. In the miR inhibitor transfection, 0.1 μL of miR inhibitor (100 μM stock) and 0.3 μL of iN-fect *in vitro* transfection reagent (INtRON Biotechnology, Gyeonggi-do, South Korea) were mixed for each well. Samples were incubated at room temperature for 15–20 min. After incubation, the mixture was made up to 100 μL with a nonserum medium added to each well. Then, 100 μL of the sample was added dropwise onto the cells from which the serum medium was removed.

### MTT Cell Viability Assay

Following 24 h of transfection, the cells were infected with the virus and incubated for 72 h. Then, the media was removed, and fresh media containing MTT (Merck, U.K.) was added; the cells were incubated at 37 °C for 4 h. Isopropanol was added to dissolve the formazan crystals. The absorbance was determined at 570 nm using a plate reader (BMG Labtech GmbH, Offenburg, Germany), and cytopathic effects were analyzed.

### miRseq and Bioinformatics Analysis

Following the infection with SARS-CoV-2 and anti-miR-1307 treatment, cells were incubated for 72 h, and total RNA was isolated. Total RNA (2 mg per sample in triplicate) was kept in RNA stable tubes (Biomatrica, Sigma-Aldrich, MO, USA) and transferred to perform miR sequencing at Genewiz (Essex, UK). According to the manufacturer’s instructions, the immediate recovery of the total RNA was performed from the tubes, and all samples were run for small RNA seq using the Illumina small RNA library to read 2 × 150 bp with approximately 10 M PE reads/sample. Our group received Genewiz (Essex, UK) data as fastq files and analyzed it on a local Linux machine running Ubuntu 20.04. Samples were analyzed using ROSALIND (https://rosalind.bio/), a tool to evaluate the cluster-based comparison between treatment groups. Data were analyzed by ROSALIND with a HyperScale architecture developed by ROSALIND, Inc. (San Diego, CA), as mentioned in our previous study.^18^ We ran a meta-analysis between the groups (Group 1: SARS-CoV-2 infected vs noninfected Vero cells; Group 2: anti-miR-1307 treated SARS-CoV-2 infected cells vs anti-miR-1307 treated Vero cells).

Following ROSALIND analysis, reads were trimmed using cutadapt1. Quality scores were assessed using FastQC2. Reads were aligned to the *Homo sapiens* genome build hg19 using bowtie3 for RNA classification and miRDeep24 for mature miRNA expression analysis. Individual bowtie-aligned sample reads were quantified using HTseq5 against Ensembl genes6. miRDeep2 alignments were normalized via Relative Log Expression (RLE) using DESeq2 R library7. DEseq2 was also used to calculate fold changes and *p*-values and perform optional covariate correction. Clustering genes for the final heat map of differentially expressed genes was done using the PAM (Partitioning Around Medoids) method and the FPC R library8. The top targeted gene predictions validated genes, and related drugs and diseases were analyzed using the multiMiR R library9. miRNA secondary structures were calculated and visualized using the ViennaRNA software10.

## Supporting Information Available

Figure S1: SARS-CoV-2 cytopathic effect, titration, and characterization; Figure S2: KEGG and GO pathways for altered miRs following anti-miR-1307-3p treatment; Figure S3: predicted targets for miR-1307-3p using Chair for Bioinformatics at the University of Saarland; Figure S4: KEGG and GO pathways for downregulated miRs between Group 1 and Group 2; Figure S5: KEGG and GO pathways for downregulated miRs between Group 1 and Group 2; Table S1: accession numbers of variant genomes (PDF)

## Notes

The authors confirm that the data supporting the findings of this study are available within the Article and its Supporting Information.
