# Supplementary material for: microRNA 1307 Is a Potential Target for SARS-CoV-2 Infection: An in Vitro Model
Source: ACS Omega. 2022 Oct 11;7(42):38003–14. doi: 10.1021/acsomega.2c05245 (PMC9578367; doi:10.1021/acsomega.2c05245)
Supplement: Supplementary file 1 — ao2c05245_si_001.pdf [file ao2c05245_si_001.pdf]

microRNA 1307 is a potential target for SARS-CoV-2 infection: an *in vitro* model

Elif Damla Arisan[1], D. Alwyn Dart[2], Guy H. Grant[3], Andrew Dalby[4], Derya Dilek Kancagi[5], Raife Dilek Turan[5,6] Bulut Yurtsever[5], Gozde Sir Karakus[5], Ercument Ovali[5], Sigrun Lange[7] and Pinar Uysal-Onganer[8]*

[1] Gebze Technical University, Institute of Biotechnology, Gebze, 41400 Kocaeli, Turkey

[2] Institute of Medical and Biomedical Education, St George's University of London, Cranmer Terrace, Tooting, London SW17 0RE, UK

[3] School of Life Sciences, University of Bedfordshire, Park Square, Luton, LU1 3JU, UK

[4] School of Life Sciences, University of Westminster, London, W1W 6UW, U.K.

[5] Acibadem Labcell Cellular Therapy Laboratory, Istanbul, 34457, Turkey

[6] Yeditepe University, Institute of Biotechnology, İstanbul, 34755, Turkey

[7] Tissue Architecture and Regeneration Research Group, School of Life Sciences, University of Westminster, London, W1W 6UW, U.K

[8] Cancer Research Group, School of Life Sciences, University of Westminster, London, W1W 6UW, UK;

* Correspondence: Pinar Uysal-Onganer

Supplemental Figures and Tables

Figure S1. SARS-CoV-2 Cytopathic effect, titration, and characterisation.

Figure S2. KEGG and GO pathways for altered miRs following anti-miR-1307-3p treatment.

Figure S3. Predicted targets for miR-1307-3p.

Figure S4. KEGG and GO pathways for downregulated miRs between Group I and Group II.

Figure S5. KEGG and GO pathways for downregulated miRs between Group I and Group II.

Table S1: Accession numbers of variant genomes.

Figure S1

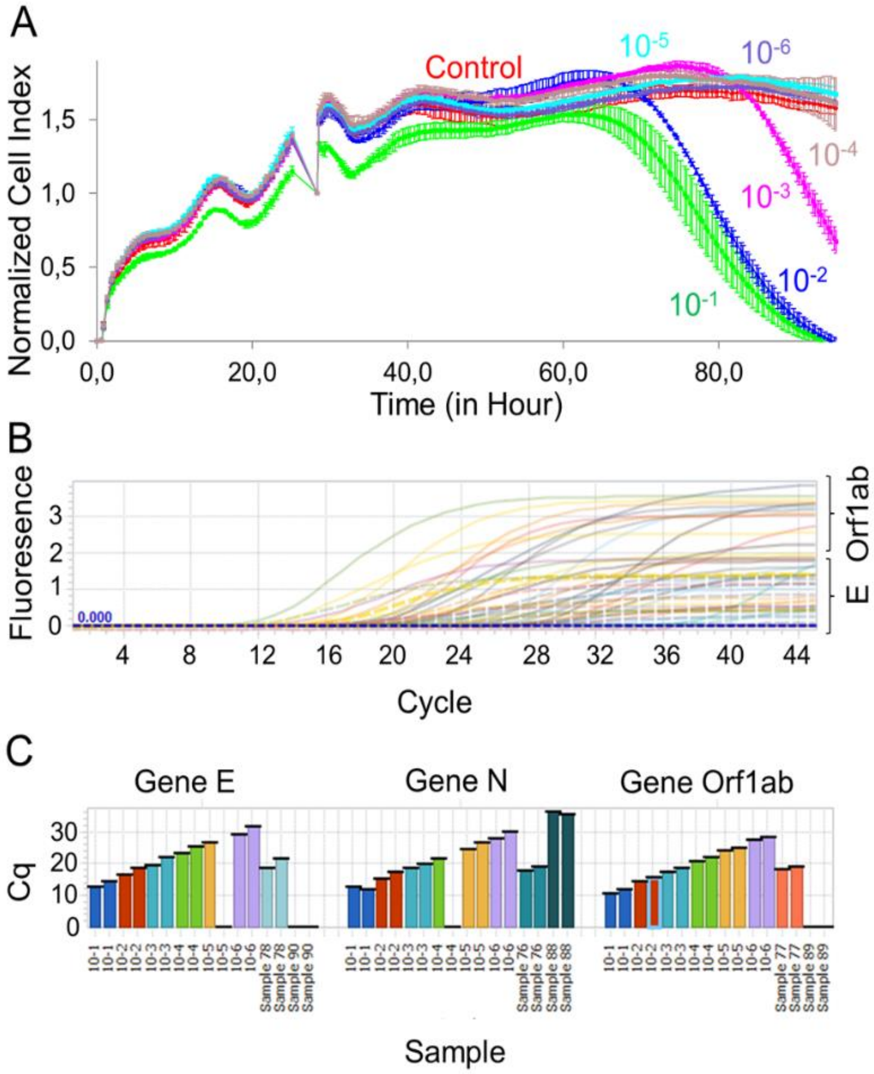

Figure S1. SARS-CoV-2 Cytopathic effect, titration, and characterisation. A. Real-time cell analysing graph showing normalised cell index value of the viable and proliferating cells. The SARS-CoV-2 virus was inoculated with the Vero E6 cells in different dilutions (10^{-1}, 10^{-2}, 10^{-3}, 10^{-4}, 10^{-5}, 10^{-6}, and no virus control) for 96 h. **B.** Representative figure of qRT-PCR amplification curves of the titrated SARS-CoV-2 samples specifically amplified with SARS-CoV-2 Orf1ab, N, and E gene primers. **C.** Cq determination and quantified value of the SARS-CoV-2 samples in different dilutions. Samples 76, 77, and 78 were synthetic normalised SARS-CoV-2 control. Samples 88, 89, and 90 were negative controls.

Figure S2

A

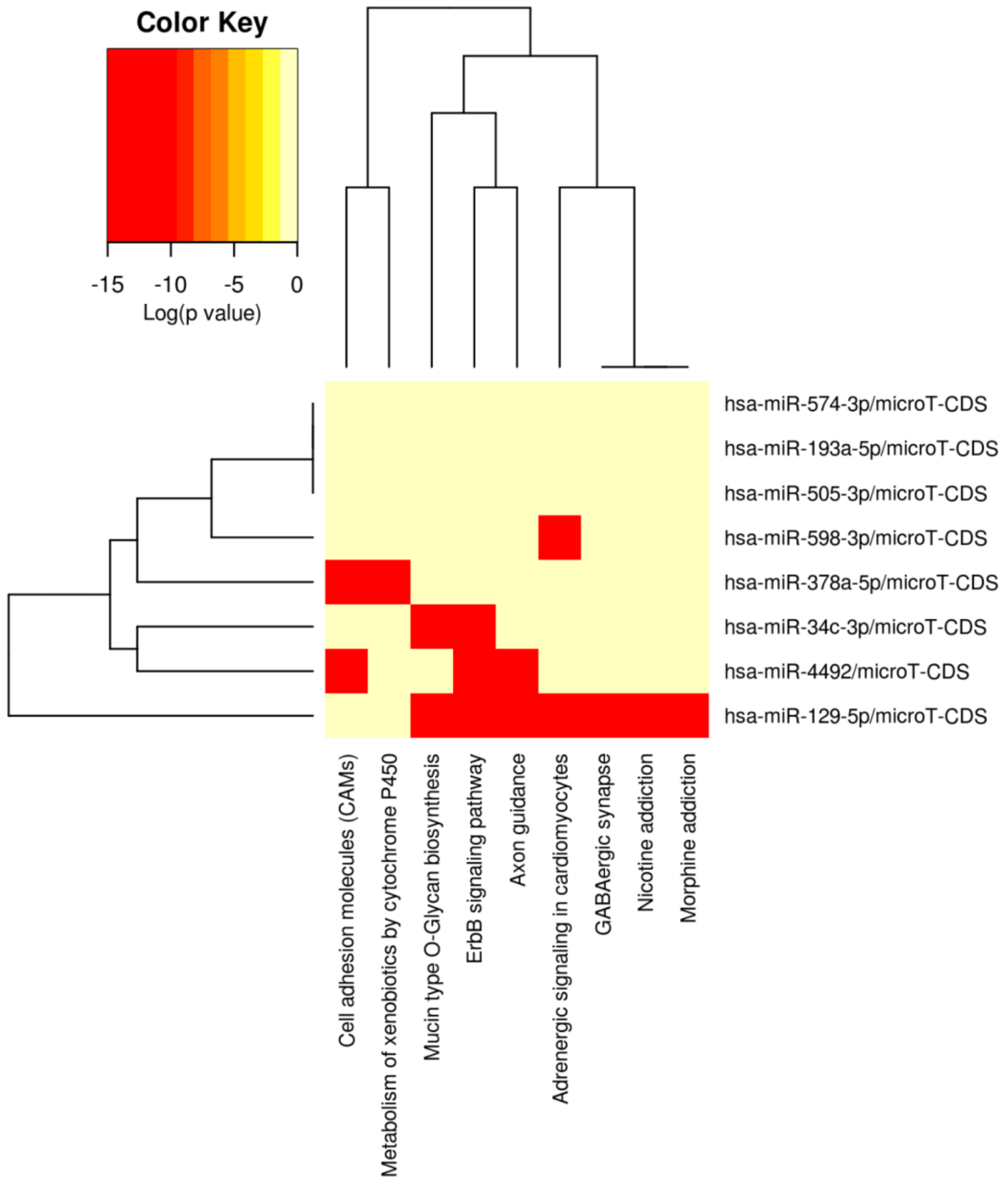
Color Key
-15
-10
-5
0
Log(p value)
hsa-miR-574-3p/microT-CDS
hsa-miR-193a-5p/microT-CDS
hsa-miR-505-3p/microT-CDS
hsa-miR-598-3p/microT-CDS
hsa-miR-378a-5p/microT-CDS
hsa-miR-34c-3p/microT-CDS
hsa-miR-4492/microT-CDS
hsa-miR-129-5p/microT-CDS
Cell adhesion molecules (CAMs)
Metabolism of xenobiotics by cytochrome P450
Mucin type O-Glycan biosynthesis
ErbB signaling pathway
Axon guidance
Adrenergic signaling in cardiomyocytes
GABAergic synapse
Nicotine addiction
Morphine addiction

Figure S2 (continued)

B

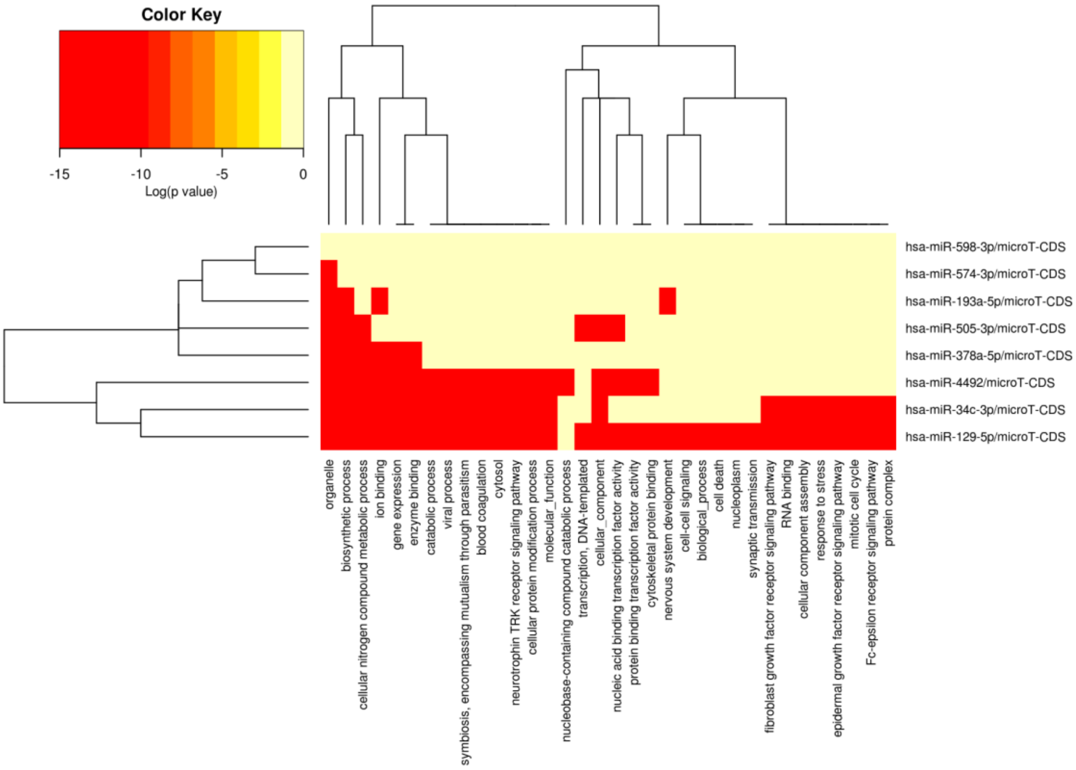

Figure S2. KEGG and GO pathways for altered miRs following anti-miR-1307-3p treatment. Downregulated top-ten miRs are shown following anti-miR-1307-3p treatment, compared to untreated control Vero cells. (**A**) KEGG **(B)** GO pathway-related alterations were shown as heat maps. $p<0.05$, MicroT threshold 0.8, Fisher Exact test hypergeometric distribution for targeted pathways clusters/heatmap drawn by DIANA-miRPath v3. 0.

Figure S3

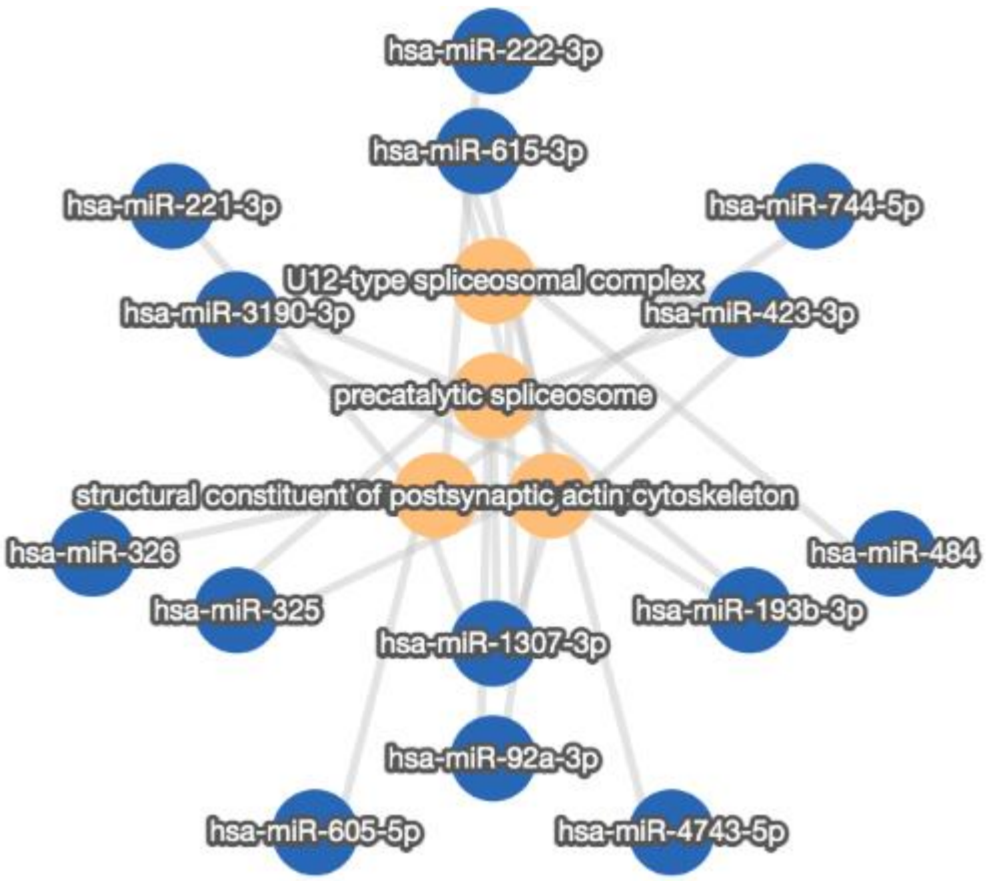

Figure S3. Predicted targets for miR-1307-3p using Chair for Bioinformatics at the University of Saarland. https://ccb-web.cs.uni-saarland.de/mirtargetlink/network.php?type=miRNA&qval=hsa-miR-1307-3p.

Figure S4

A

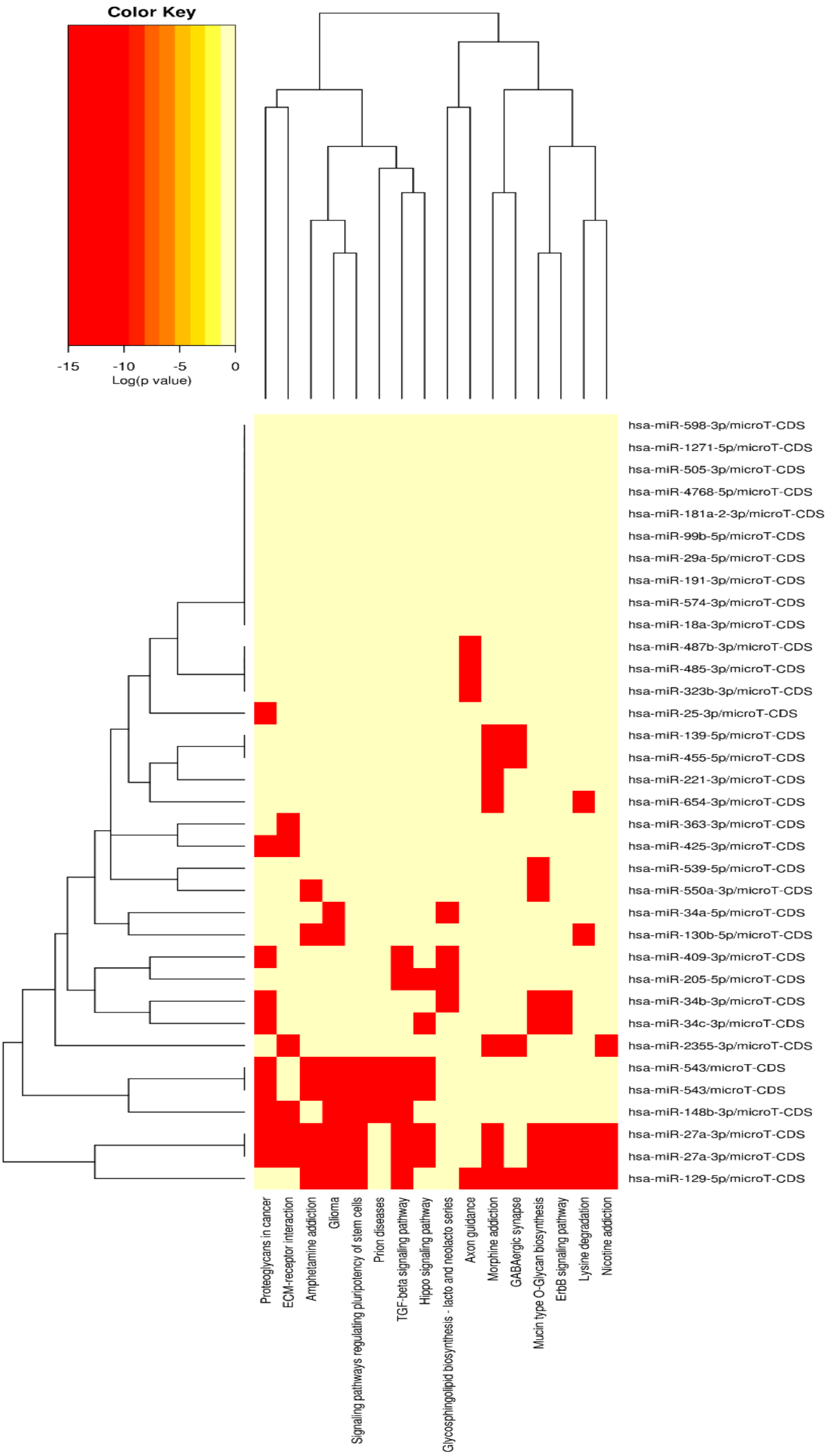
Color Key
-15
-10
-5
0
Log(p value)
hsa-miR-598-3p/microT-CDS
hsa-miR-1271-5p/microT-CDS
hsa-miR-505-3p/microT-CDS
hsa-miR-4768-5p/microT-CDS
hsa-miR-181a-2-3p/microT-CDS
hsa-miR-99b-5p/microT-CDS
hsa-miR-29a-5p/microT-CDS
hsa-miR-191-3p/microT-CDS
hsa-miR-574-3p/microT-CDS
hsa-miR-18a-3p/microT-CDS
hsa-miR-487b-3p/microT-CDS
hsa-miR-485-3p/microT-CDS
hsa-miR-323b-3p/microT-CDS
hsa-miR-25-3p/microT-CDS
hsa-miR-139-5p/microT-CDS
hsa-miR-455-5p/microT-CDS
hsa-miR-221-3p/microT-CDS
hsa-miR-654-3p/microT-CDS
hsa-miR-363-3p/microT-CDS
hsa-miR-425-3p/microT-CDS
hsa-miR-539-5p/microT-CDS
hsa-miR-550a-3p/microT-CDS
hsa-miR-34a-5p/microT-CDS
hsa-miR-130b-5p/microT-CDS
hsa-miR-409-3p/microT-CDS
hsa-miR-205-5p/microT-CDS
hsa-miR-34b-3p/microT-CDS
hsa-miR-34c-3p/microT-CDS
hsa-miR-2355-3p/microT-CDS
hsa-miR-543/microT-CDS
hsa-miR-543/microT-CDS
hsa-miR-148b-3p/microT-CDS
hsa-miR-27a-3p/microT-CDS
hsa-miR-27a-3p/microT-CDS
hsa-miR-129-5p/microT-CDS
Proteoglycans in cancer
ECM-receptor interaction
Amphetamine addiction
Glioma
Signaling pathways regulating pluripotency of stem cells
Prion diseases
TGF-beta signaling pathway
Hippo signaling pathway
Glycosphingolipid biosynthesis - lacto and neolacto series
Axon guidance
Morphine addiction
GABAergic synapse
Mucin type O-Glycan biosynthesis
ErbB signaling pathway
Lysine degradation
Nicotine addiction

Figure S4 (continued)

B

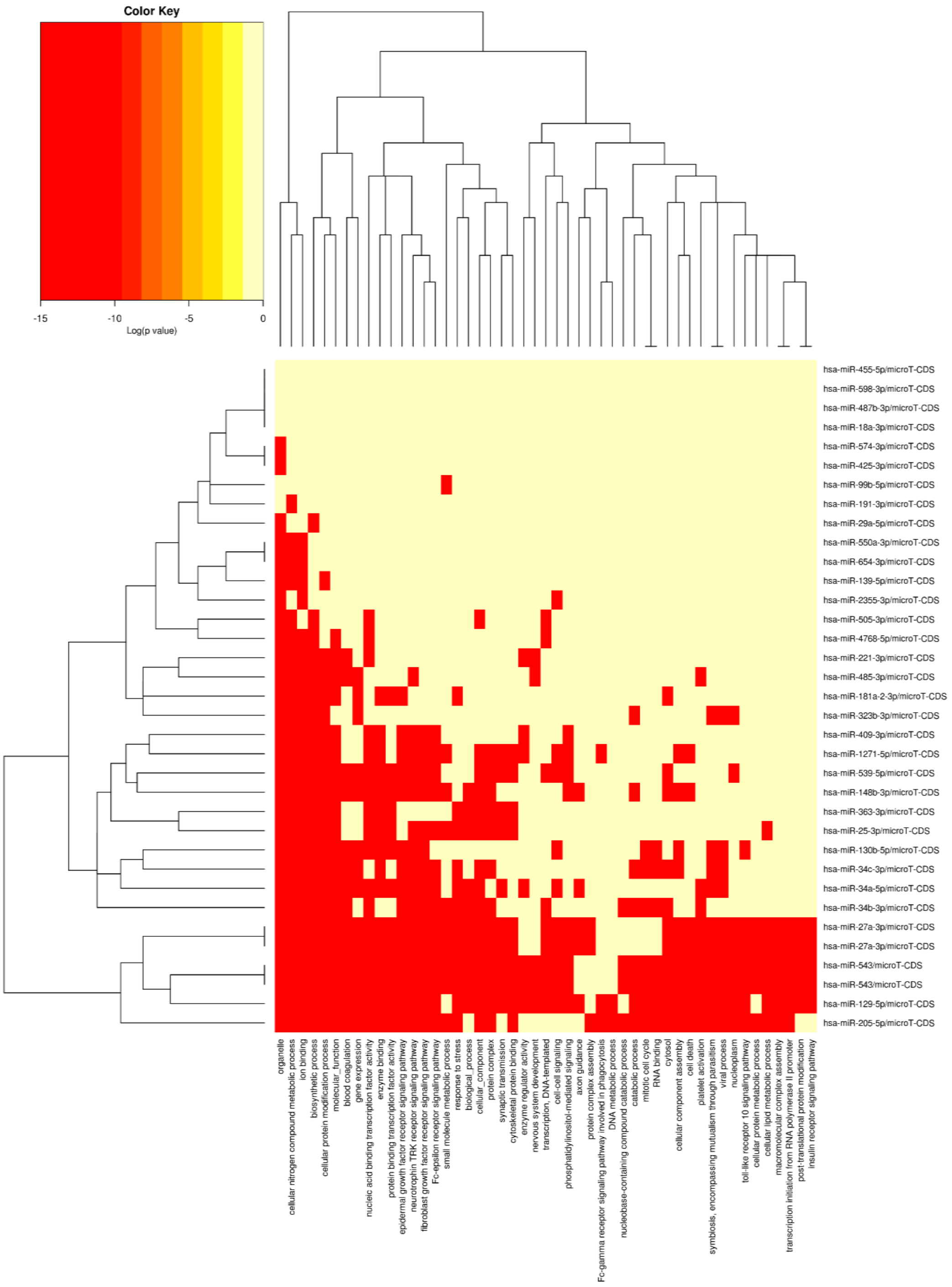

Figure S4. KEGG and GO pathways for downregulated miRs between Group 1 and Group 2. Downregulated top miRs were shown following anti-miR-1307-3p treatment compared to untreated control Vero cells. (**A**) KEGG (**B**) GO pathway-related alterations were shown as heat maps. $p<0.05$, MicroT threshold 0.8, Fisher Exact test hypergeometric distribution for targeted pathways clusters/heatmap drawn by DIANA-miRPath v3.0.

Figure S5

A

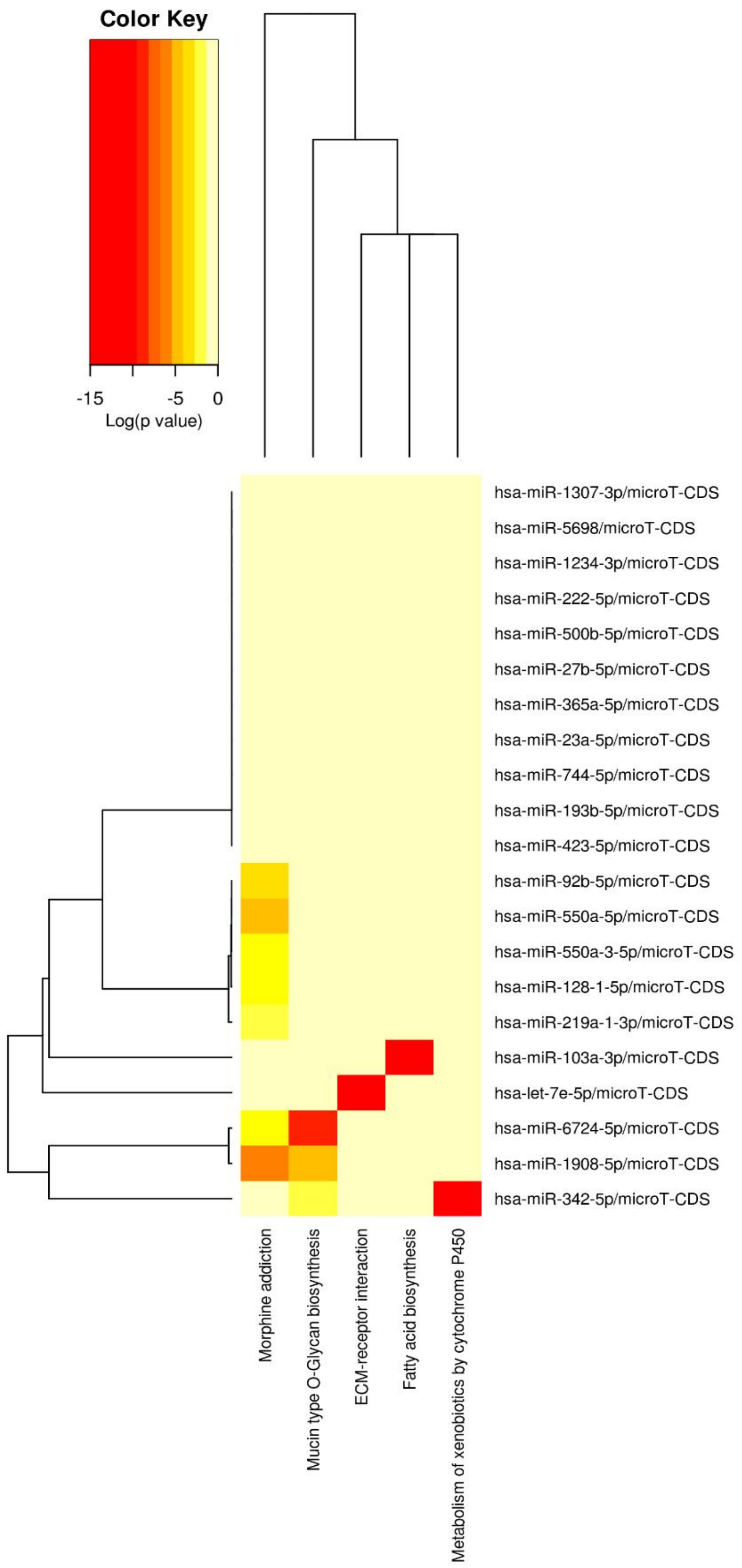
Color Key
-15
-5
0
Log(p value)
hsa-miR-1307-3p/microT-CDS
hsa-miR-5698/microT-CDS
hsa-miR-1234-3p/microT-CDS
hsa-miR-222-5p/microT-CDS
hsa-miR-500b-5p/microT-CDS
hsa-miR-27b-5p/microT-CDS
hsa-miR-365a-5p/microT-CDS
hsa-miR-23a-5p/microT-CDS
hsa-miR-744-5p/microT-CDS
hsa-miR-193b-5p/microT-CDS
hsa-miR-423-5p/microT-CDS
hsa-miR-92b-5p/microT-CDS
hsa-miR-550a-5p/microT-CDS
hsa-miR-550a-3-5p/microT-CDS
hsa-miR-128-1-5p/microT-CDS
hsa-miR-219a-1-3p/microT-CDS
hsa-miR-103a-3p/microT-CDS
hsa-let-7e-5p/microT-CDS
hsa-miR-6724-5p/microT-CDS
hsa-miR-1908-5p/microT-CDS
hsa-miR-342-5p/microT-CDS
Morphine addiction
Mucin type O-Glycan biosynthesis
ECM-receptor interaction
Fatty acid biosynthesis
Metabolism of xenobiotics by cytochrome P450

Figure S5 (continued)

B

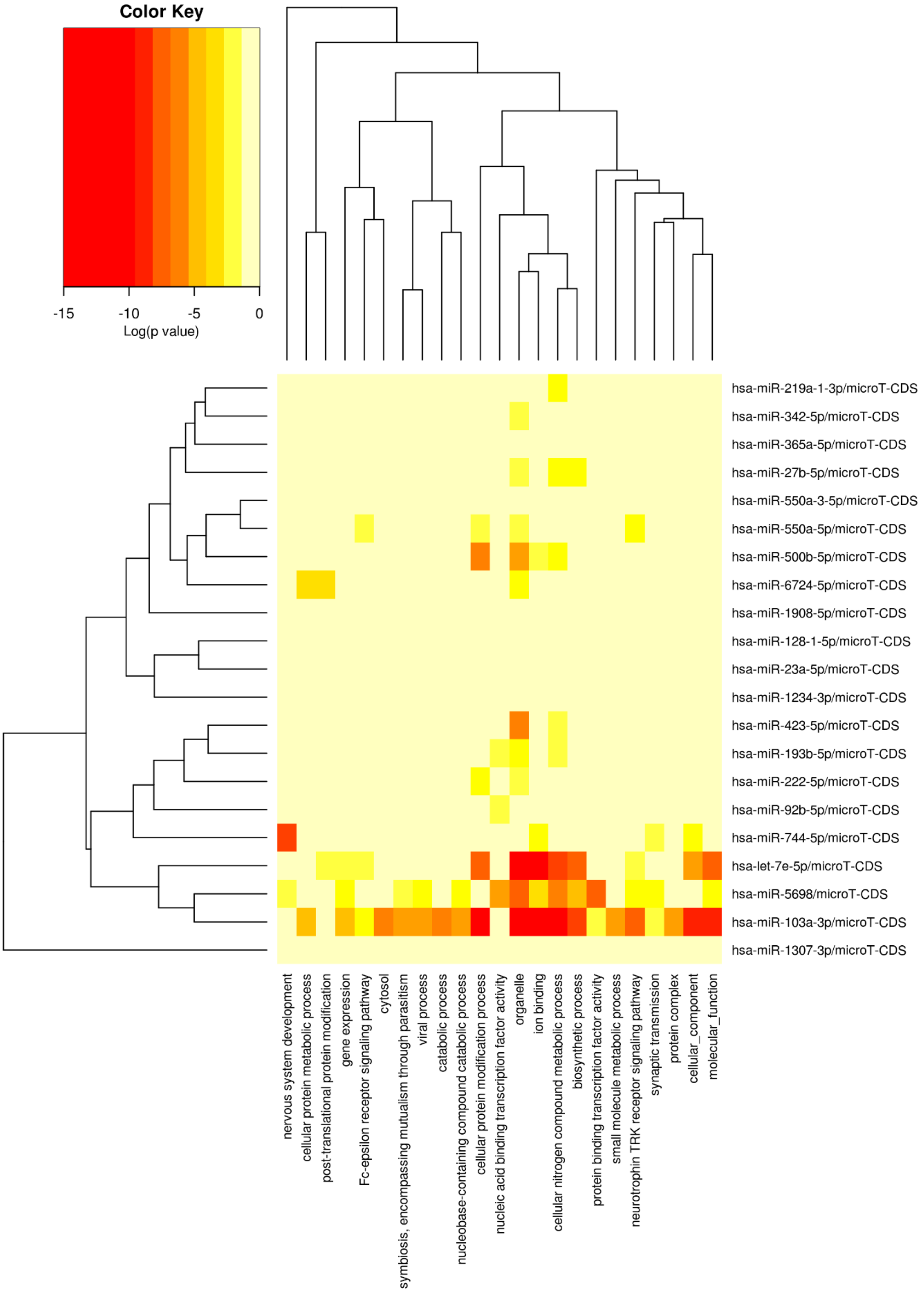

Figure S5. KEGG and GO pathways for upregulated miRs between Group 1 and Group 2. Upregulated top miRs were shown following anti-miR-1307-3p treatment compared to untreated control Vero cells. (**A**) KEGG (**B**) GO pathway-related alterations were shown as heat maps. $p<0.05$, MicroT threshold 0.8, Fisher Exact test hypergeometric distribution for targeted pathways clusters/heatmap drawn by DIANA-miRPath v3.0.

Table S1

Table S1: Accession numbers of variant genomes. Full genome sequence alignments were carried out using the Clustal Omega server at EBI, Hinxton https://www.ebi.ac.uk/Tools/msa/clustalo/).

Sequence name	GenBank Accession number
OC43	AY391777.1
HKU1	AY884001.1
NL63	MG772808.1
229E	MF542265.1
MERS	KT029139.1
SARS	AY278488
Omicron BA.2	ON058043.1
Delta	MZ359841.1
Omicron BA.1	OL672836.1
Gamma	MZ169911.1
Beta	MW598419.1
Alpha	MZ344997.1
WH-1	NC_045512.2
RatG13	MN996532.2
